# Recombinant DNA the Bio-Revolution, Between Promise, Hurdles, and Achievements

**DOI:** 10.3390/ijms26146611

**Published:** 2025-07-10

**Authors:** Mohamed Raafat El-Gewely

**Affiliations:** Department of Medical Biology, Faculty of Health Sciences, UiT Arctic University of Tromsø, 9037 Tromsø, Norway; raafat.el-gewely@uit.no

Not too long after the discovery of restriction enzymes, by Warner Arbor group in 1962 and 1963 [1,2] followed by the work of Hamilton Smith [3]; Dan Nathan and Hamilton Smith [4]; Wil A. M. Loenen et al. 2014 [5], elegantly reviewed the restriction enzyme type I discovery and history. Noting that Werner Arber, Dan Nathans, and Hamilton Smith, received the Nobel prize in 1978.

In 1973 Stanley Cohen, Anita Chang, Herbert Boyer, and Robert Helling published their first attempt to use restriction enzymes to cut pieces of DNA, ligate it to a plasmid and transform the ligated complex into the *E. coli* host cells, termed the recombinant DNA process. No Nobel prize for this milestone [6].

The first therapeutic recombinant protein on the market was insulin engineered by Genentech in 1978 and further developed and produced on a large scale by Elli Lilly as (Humulin). It was on the market after 5 months’ evaluation by FDA on 28 October 1982.

Currently the number of recombinant therapeutic proteins on the market has significantly increased (Recombinant Proteins|DrugBank Online,) and so is its projection (URL last accessed 27 June 2025). The expectations of therapeutic recombinant proteins market are huge. The market, estimated at around USD 3.97 billion in 2025, and is forecasted to hit around USD 11.32 billion by 2034 (https://www.precedenceresearch.com/recombinant-proteins-market) (URL last accessed on 27 June 2025). Noting that a parallel market of biosimilars is thriving.

There are several problems in expressing an active desired recombinant protein whether as therapeutic or as a validated active recombinant protein target for drug discovery and development.

Most of the problems were due to lack or reduced expression of the recombinant protein or alternatively producing insoluble protein as inclusion bodies. This special issue is aiming to address such hurdles and current methods to overcome these hurdles. To name the most encountered obstacles, for example the discrepancy of the utilized amino acid tRNA codes between the heterologous host and the original organism. The codon bias in the heterologous gene expression and the original organism as reviewed by Claes Gustafsson et al. in 2004 [7]. Harmonization of the code became the standard as discussed by Evelina Angov et al., 2008 [8]. McDonald et al., 2015 reported that such discrepancy in codon utilization bias between human cells and *E. coli* cells is due to evolution during a recombinant gene expression of human genes affecting the profile of expressed tRNA expression in *E. coli* cells [9]. Interestingly, based on the data of one thousand human genome projects, Parisien et al. 2013 concluded that tRNA genes are more diverse than conventionally perceived [10]. A recurrent neural network (RNN) model of codon optimization achieved higher level of protein expression when transfected transiently or stably into Chinese hamster ovary cells [11]. Demissie et al., 2025 [12] presented an integrated approach for the interplay of multiple factors to fine-tune the genetic sequences of the desired recombinant protein to match the translational machinery and codon usage preferences of specific heterologous organism.

The belief that the primary structure/sequence of protein alone determines its tertiary structure independent of the cellular environment is behind most of the problems encountering recombinant protein production. This was based on the works of at least two Nobel laureates; Anfinsen as well as Crick. Of course, it is true, but how? Several recombinant proteins expressed as misfolded inclusion bodies, which would require in vitro manipulations to solubilize [13,14,15]. Moreover, several synthetic peptides were not active because they did not assume the proper tertiary structure and must be subsequently solubilized [16,17,18,19,20].

The numerous reported difficulties in producing active recombinant proteins lead to the conclusion by El-Gewely, 2009, that the final structure–function of proteins depends on the folding environment, and protein primary structure does not alone guarantee a unique tertiary structure, functionality, or even solubility [21]. Primary structure is a key factor for protein folding, but so is the genetic environment of the cell. As matter of fact, we have managed to compromise the primary structure of the TrpR monomer protein, by site-directed mutagenesis, yet some of these heterodimer’s combinations were active in vivo although these active heterodimers contained two complementing mutants [22]. The basic genetic formula can still apply, assuming that protein solubility and activity is the phenotype P, G is the genotype dictating the primary structure, while E = Environment factors and E/G is the interaction between environment and primary structure as well as host genes.

**P = G + E +E/G**

However, according to the books (and Anfinsen conclusions) by ignoring the cell environment and its relevant genetics influences: **P = G**

And that could explain most of the observed difficulties.

The co-expression of chaperones together with target recombinant protein in the heterologous host, Improvement of the recombinant protein production, both in quantity and activity [23,24,25].

Also, by screening synthetic signal peptide library, Jeon et al., 2024 achieved improved production and secretion of target recombinant protein using *Corynebacterium glutamicum* as the heterologous host [26]. See selected slide from one of my presentations on the subject at Bayer Berlin 1n 2015: Bayer-Berlin 7-20-2015-Selected slides.pptx.

It is very crucial to have active and soluble recombinant proteins to produce therapeutic recombinant proteins. Once a protein validated to be a drug discovery target, it is equally important to produce physical amounts of such recombinant protein for the use in drug discovery and development process. McGonigle, 2024, reviewed the subject of drug discovery using recombinant proteins [27].

In recent years, some efforts in using the mRNA directly replace the therapeutic recombinant protein directly instead of going through the efforts of expressing the active therapeutic protein in a heterologous host [28,29].

Interesting recent development is dealing with the prediction of the tertiary protein structure only from its amino acid sequence, as reviewed recently by Meng et al., 2025 [30], thus reducing several required steps in drug discovery, including the expression of active and soluble recombinant protein target for the drug discovery, crystallization of the protein and conducting the essential X-ray crystallography. This would facilitate drug discovery by adding a valuable tool, but this will not impact on the therapeutic recombinant protein field, or in producing active antibodies against undesired expressed protein.
